# Age Incidence, Sex, and Comparative Frequency in Disease

**Published:** 1910-06

**Authors:** 


**Age Incidence, Sex, and Comparative Frequency in Disease.** By JAMES GRANT ANDREW, M.B., M.S. Pp. xix, 439. London : Baillière, Tindall & Cox. 1909.

If we knew more about the circumstances under which predisposition to disease arises, the problem of prevention—which is the highest aim of modern medicine—would be simplified.

In recent times pathologists have been so fully occupied with the search for particulate causes of disease in the realm of the infinitely minute, that the great importance of predisposition, without which particulate causes of disease are comparatively speaking inoperative, has been obscured and overlooked.

Among the predisposing causes of disease, age and sex are important factors. The time is now ripe for the scientific study of this part of the subject, regard being had for generalised considerations, as well as for items of detail. In this sense there is here an opening for pioneers, since almost everything remains to be done.

The census reports, the annual reports of the Registrar-General, the reports of the Lunacy and Prison Commissioners, and other publications of this kind, will furnish valuable material.

The annual reports of the registrars of such hospitals as have for a series of years published detailed analyses of their cases, and specialised monographs dealing with this aspect of the subject, may also be relied on for furnishing useful data.

It is with this latter class of publication that Dr. J. G. Andrew's work must be classed.

This is the age of detail, and Dr. Andrew has made no attempt to overpass it by rising from details to generalised considerations.

His work is a statistical analysis of 42,603 consecutive cases under treatment at the Glasgow Western Infirmary, so as to show the age and sex incidence of disease, as well as its comparative frequency.

Although the gross total of cases seems large, yet when the items are split up under their various headings the numbers are nearly always too small to furnish reliable averages.

This is, of course, a serious shortcoming, and it is owing to this lack of "massif" in the totals that the analyses often seem to be over-elaborated.

The work would have been of greater scientific value if the analysis had been based on at least 250,000 cases, and this might well have been effected if Dr. Andrew had availed himself of the records of other hospitals, and of monographs in which large numbers of cases of this kind have been massed and analysed.

The future investigator, who will doubtlessly make this his task, will have reason to be grateful to Dr. Andrew for having facilitated a stage of his arduous undertaking by the publication of the present valuable statistical compilation.

